# PROX1 is a novel pathway-specific prognostic biomarker for high-grade astrocytomas; results from independent glioblastoma cohorts stratified by age and IDH mutation status

**DOI:** 10.18632/oncotarget.11957

**Published:** 2016-09-10

**Authors:** Kenney R. Roodakker, Tamador Elsir, Per-Henrik D. Edqvist, Daniel Hägerstrand, Joseph Carlson, Malgorzata Lysiak, Roger Henriksson, Fredrik Pontén, Johan Rosell, Peter Söderkvist, Roger Stupp, Elena Tchougounova, Monica Nistér, Annika Malmström, Anja Smits

**Affiliations:** ^1^ Department of Neuroscience, Neurology, Uppsala University, University Hospital, Uppsala, Sweden; ^2^ Department of Oncology-Pathology, Karolinska Institutet, Cancer Center Karolinska R8:05, Karolinska University Hospital, Stockholm, Sweden; ^3^ Department of Immunology, Genetics and Pathology, Uppsala University, Rudbeck Laboratory, Uppsala, Sweden; ^4^ Science for Life Laboratory, Uppsala University, Uppsala, Sweden; ^5^ Department of Cell Biology, Linköping University, Linköping, Sweden; ^6^ Department of Radiation Sciences & Oncology, Umeå University Hospital, Umeå, Sweden; ^7^ Regional Cancer Center, Stockholm Gotland, Stockholm, Sweden; ^8^ Regional Cancer Center South East Sweden and Department of Clinical and Experimental Medicine, Linköping University, Linköping, Sweden; ^9^ Department of Advanced Home Care and Department of Clinical and Experimental Medicine, Linköping University, Linköping, Sweden; ^10^ Department of Oncology, University Hospital Zurich, Zurich, Switzerland; ^11^ Department of Clinical Neuroscience and Rehabilitation, Institute of Neuroscience and Physiology, Sahlgrenska Academy, University of Gothenburg, Gothenburg, Sweden

**Keywords:** PROX1, malignant astrocytomas, IDH mutations, primary glioblastomas, secondary glioblastomas, Gerotarget

## Abstract

PROX1 is a transcription factor with an essential role in embryonic development and determination of cell fate. In addition, PROX1 has been ascribed suppressive as well as oncogenic roles in several human cancers, including brain tumors. In this study we explored the correlation between PROX1 expression and patient survival in high-grade astrocytomas. For this purpose, we analyzed protein expression in tissue microarrays of tumor samples stratified by patient age and *IDH* mutation status. We initially screened 86 unselected high-grade astrocytomas, followed by 174 IDH1-R132H1 immunonegative glioblastomas derived from patients aged 60 years and older enrolled in the Nordic phase III trial of elderly patients with newly diagnosed glioblastoma. Representing the younger population of glioblastomas, we studied 80 *IDH*-wildtype glioblastomas from patients aged 18-60 years. There was no correlation between PROX1 protein and survival for patients with primary glioblastomas included in these cohorts. In contrast, high expression of PROX1 protein predicted shorter survival in the group of patients with *IDH*-mutant anaplastic astrocytomas and secondary glioblastomas. The prognostic impact of PROX1 in *IDH*-mutant 1p19q non-codeleted high-grade astrocytomas, as well as the negative findings in primary glioblastomas, was corroborated by gene expression data extracted from the Cancer Genome Atlas. We conclude that PROX1 is a new prognostic biomarker for 1p19q non-codeleted high-grade astrocytomas that have progressed from pre-existing low-grade tumors and harbor *IDH* mutations.

## INTRODUCTION

Glioblastomas are the most common and lethal type of adult primary brain tumors [1,2]. Mean survival for patients with glioblastoma is around 15 months [3]. Traditionally, glioblastomas are separated into two major classes; primary glioblastomas that arise *de novo* in predominantly older patients and secondary glioblastomas in younger patients with a history of prior low-grade gliomas. Recent advances in cancer genomics have provided support for distinctive molecular correlates of these two clinical phenotypes. Primary glioblastomas are characterized by *PTEN* tumor suppressor mutations/deletions, *EGFR* amplification and *TERT* promoter mutations, whereas secondary glioblastomas frequently have mutations/deletions of *IDH1/2*, *TP53* and *ATRX* [4]. In the 2016 WHO classification of brain tumors, some of these key aberrations have been added to define new tumor entities based on histological and molecular features [5]. Importantly, the identification of distinctive molecular pathways has introduced new concepts for therapeutic management, based on the clinical diversity of these tumors.

Cancer development is caused by defective regulation of cell growth, differentiation, death and/or survival. While cancer cells can inactivate elements of all these pathways, they never disable the entire signaling cascades [6]. One of the most fundamental traits of cancer cells involves their self-renewal capability and ability to sustain proliferation. Identifying aberrant expression of key regulatory proteins of these signaling pathways is an important step to unravel oncogenic mechanisms, guide therapeutic decisions and develop new biologically based therapies.

PROX1 is a transcription factor with a widespread role in cell cycle control and progenitor cell differentiation that is critical for embryonic development [7]. During development of the CNS, PROX1 regulates progenitor cell differentiation and initiation of neurogenesis, where high levels lead to depletion of the progenitor cell pool. Expression of PROX1 in the developing and adult mammalian brain corresponds to areas known to harbor neural progenitor and neural stem cells, i.e. the subventricular zone of the lateral ventricle wall and the subgranular zone in the dentate gyrus [8-10]. In addition to its role in physiological development, PROX1 has been ascribed tumor suppressive as well as oncogenic effects in human cancers [7].

We have previously shown that the proportion of PROX1 expressing tumor cells correlates with the malignancy grade of gliomas [11], and that increased PROX1 protein expression predicts shorter survival for patients with diffuse low-grade gliomas [12]. The aim of the present study was to determine the prognostic impact of PROX1 in high-grade astrocytomas. We hypothesized that PROX1 is a prognostic factor for patients with primary glioblastomas, the most common type of glioblastoma that arises in older patients and lacks *IDH1/2* mutations. Alternatively, and based on our previous findings in diffuse low-grade gliomas, PROX1 may be a prognostic marker for *IDH*-mutant astrocytomas that have progressed from pre-existing low-grade tumors [12]. For this purpose, we used tissue microarrays (TMAs) of high-grade astrocytomas representing the different pathways based on *IDH* mutation status. Since age is one of the strongest predictors of survival for patients with glioblastomas, separate analyses were performed for younger and older patients [13].

**Figure 1:**
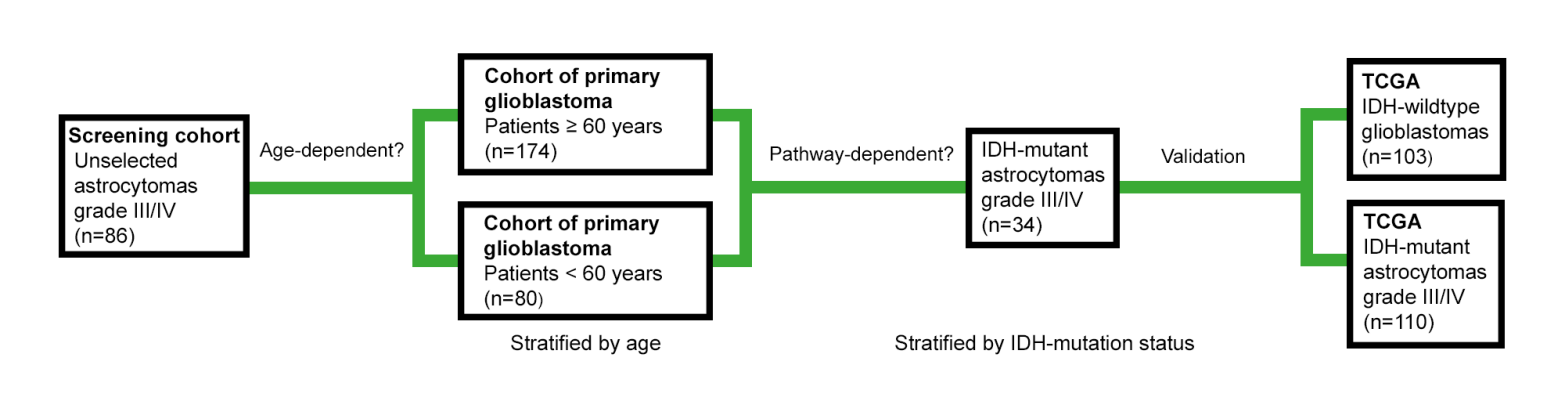
Illustration of the study design We used tissue microarrays (TMAs) to explore PROX1 protein signatures in an unselected screening cohort of astrocytomas grade III and IV, in cohorts of primary glioblastomas stratified by age and in *IDH*-mutant astrocytomas grade III and IV. In the final step, gene expression data extracted from The Cancer Genome Atlas (TCGA) in 1p19q non-codeleted astrocytomas grade III and IV that were stratified by IDH mutation status were used to corroborate the findings.

Figure 1 illustrates the study design. For screening, we used an unselected cohort of 86 adult patients with high-grade astrocytomas receiving radiotherapy (RT) at our hospital. Representing the older population of primary glioblastoma, we selected 174 IDH1-R132H1 immunonegative tumors from patients aged ≥ 60 years enrolled in the Nordic phase III trial in elderly patients with newly diagnosed glioblastomas, comparing standard RT, hypofractionated RT and temozolomide (TMZ) [14]. The first results of this TMA are presented here. Representing the younger population, 80 *IDH*-wildtype glioblastomas were derived from a treatment cohort with patients aged < 60 years receiving RT and different treatment schedules of TMZ. We found that PROX1 protein levels did not predict survival in these cohorts. In contrast, PROX1 protein expression correlated with survival in 34 *IDH*-mutant high-grade astrocytomas. In a final step, these findings were corroborated in *IDH*-wildtype glioblastomas and in *IDH*-mutant 1p19q non-codeleted high-grade astrocytomas extracted from The Cancer Genome Atlas (TCGA). The potential role of PROX1 as a pathway-specific biomarker for astrocytomas is discussed.

## RESULTS

### Unselected cohort of high-grade astrocytomas (Table 1)

#### Screening cohort of high-grade astrocytomas (*n* = 86)

A TMA was constructed from a retrospective cohort of adult patients with high-grade gliomas receiving RT at our hospital, as previously described [15]. Tumors diagnosed as astrocytomas WHO grade III and IV, based on histological classification were collected for the present study. Fifteen of the 86 tumor samples showed immunoreactivity for mutant R132H IDH1 protein (mIDH1R132) [16]. The clinical characteristics of the patients included in the screening cohort are shown in Table 1. Mean age was 57 years, mean survival 12.7 months. All patients had died at the end of the study. The parameter age, entered as continuous variable in the multivariate Cox regression model, correlated with survival, whereas gender, Karnofsky performance status (KPS ≥ 80 *versus* KPS < 80), surgery (resection *versus* biopsy), WHO malignancy grade (grade III *versus* IV) and IDH1 mutated protein (mutated *versus* non-mutated) did not affect survival (Table 1).

**Table 1:** Cox multivariate regression in high-grade astrocytomas for patients included in the screening cohort (*n* = 86)

Variables	*n*	HR (95% CI)	*p*-value
Gender (female *vs* male)	48/38	1.07 (0.68-1.69)	0.76
Age	86	1.03 (1.01-1.05	0.0038*
Performance status (KPS ≥80 *vs* <80)	71/15	0.94 (0.52-1.68)	0.83
Surgery (resection *vs* biopsy)	81/5	1.20 (0.43-3.37)	0.72
Malignancy grade (WHO III *vs* IV)	19/67	0.66 (0.35-1.23)	0.19
IDH1 (mutation *vs* no mutation)	15/71	0.76 (0.38-1.52)	0.44
PROX1 protein (<50%; 50-90%; >90%)	8/41/37	1.12 (0.76-1.67)	0.56

HR= hazard ratio; CI = confidence interval;

* *p* ≤ 0.05

#### Immunostaining of PROX1

**Figure 2:**
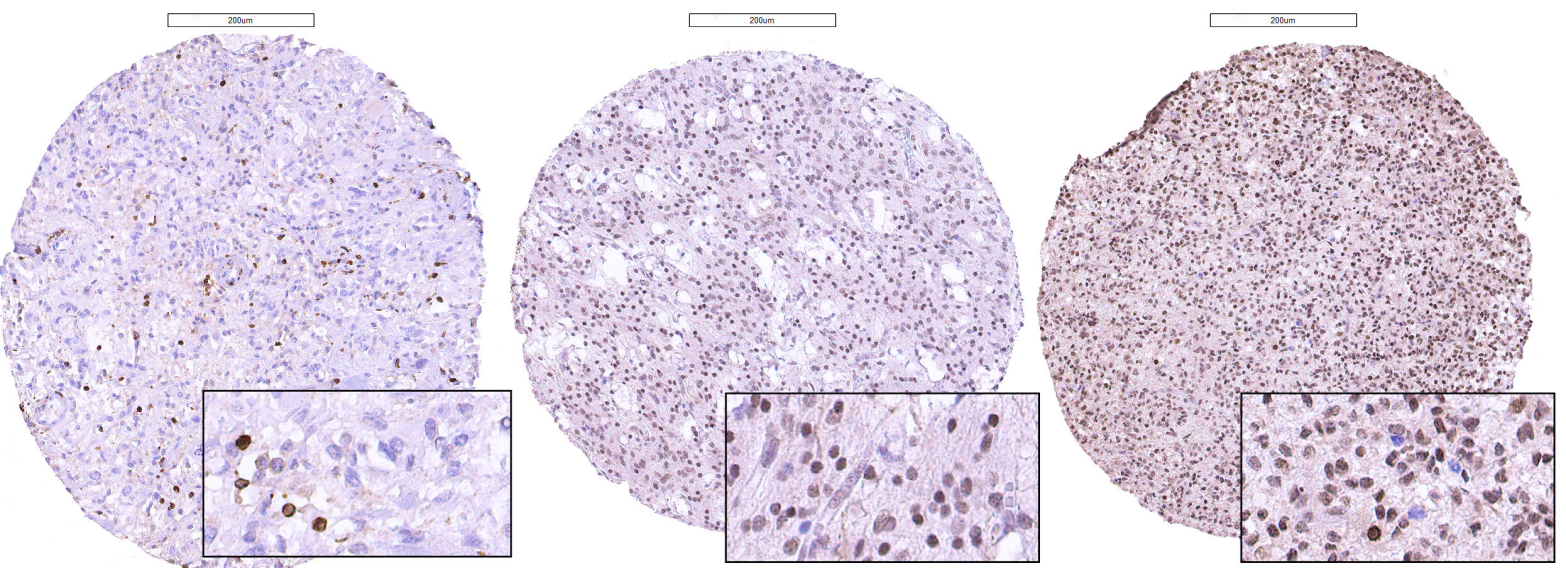
Photomicrographs of immunostaining with anti-PROX1 antibodies in glioblastomas, illustrating the scoring of the proportion of immunoreactive cells set as: ≤ 50% (left), 50-90% (middle) or ≥ 90% (right) of the total amount of tumor cells

PROX1 protein was localized in the nucleus of the tumor cells. Immunopositive tumor cells for the anti-PROX1 antibody were detected in all samples. Protein expression was scored as 1+ (< 50% positive tumor cells), 2+ (50-90% positive tumor cells) or 3+ (≥ 90% positive tumor cells), as illustrated in Figure 2. Table 1 shows the distribution of PROX1 protein in the different tumor samples included in the TMA. In general, PROX1 was widely expressed in the majority of tumors. Eight samples were scored with less than 50% positive cells, 41 samples were scored with 50-90% positive cells, and 37 samples had more than 90% positive cells (Table 1).

#### Screening cohort: survival by PROX1 protein expression

As shown in Table 1, age was a statistically significant prognostic factor whereas PROX1 was not identified as a predictor for survival in the Cox regression model (*p* = 0.56). These findings in the screening cohort prompted us to explore the correlation between PROX1 protein expression and survival in two age-specific cohorts of glioblastoma, the results of which are presented in detail below.

### Selected cohorts of primary glioblastomas (Table 2)

#### Elderly patients with newly diagnosed glioblastomas (*n* = 174)

A TMA was generated from patients aged 60 years or older with glioblastoma enrolled in the Nordic trial of elderly patients with newly diagnosed glioblastomas [14]. This TMA consisted of 175 surgical samples from the original 342 randomized patients, selected based on the availability of tumor tissues and on informed consent to use samples for translational studies at the local centers. For the present study, we excluded one tumor with immunoreactivity for the mutated IDH1 protein (mIDH1R132), leaving a total number of 174 tumors [16]. DNA sequencing of *IDH1/2* mutations was not performed. The clinical and molecular characteristics of the 174 elderly patients with IDH1-immunonegative glioblastomas are shown in Table 2a. Mean age was 69.3 years, mean survival was 10.4 months (range 0.4-126 months). At the time of last follow-up (1^st^ January 2011), 169 patients had died, 3 were alive, and 2 were lost to follow-up. Multivariate survival analysis by Cox regression identified WHO Performance Scale (0-1 *versus* 2-3), *MGMT* promoter methylation status (methylated *versus* unmethylated) and steroids at baseline (yes *versus* no) as independent prognostic factors, while surgery (resection *versus* biopsy), age (continuous) and treatment arm (standard / hypofractionated RT *versus* TMZ) did not reach significance (Table 2a).

#### Survival by PROX1 in elderly patients with newly diagnosed glioblastomas

The distribution of PROX1 protein expressing cells in the tumors is shown in Table 2a. As for the screening cohort, PROX1 protein levels were generally high. There was no correlation between PROX1 protein expression and survival in the multivariate Cox regression model (*p* = 0.28) (Table 2a). Similar negative results were found for the three treatment arms separately (data not shown). There were no statistically significant pairwise interactions between PROX1 and the other variables in the model.

**Table 2a:** Multivariate Cox regression in primary glioblastomas for patients 60 years and older (*n* = 174)

First line Treatment Variables	Standard RT (*n* = 49)	Hypo RT (*n* = 58)	TMZ (*n* = 67)	HR^#^ (95% CI)	*p* -value
Gender (female *vs* male)	14/35	32/26	25/42	0.79 (0.54-1.15)	0.22
Age (60-70 *vs* > 70)	34/15	34/24	36/31	1.08 (0.75-1.55)	0.68
WHO Performance Status (0-1 *vs* 2-3)	36/13	48/10	51/16	0.52 (0.33-0.81)	0.004*
Surgery type (resection *vs* biopsy)	44/5	54/4	60/7	0.57 (0.30-1.08)	0.08
Taking steroids at baseline (no *vs* yes)	23/25 (1 m)	31/27	35/31 (1 m)	0.51 (0.35-0.74)	0.0004*
Radiotherapy (standard + hypo *vs* TMZ)	107	67	0.92 (0.64-1.32)	0.65	
MGMT status (methyl *vs* unmethyl)	15/29 (5 m)	25/25 (8 m)	22/36 (9 m)	0.69 (0.48-0.99)	0.047*
PROX1 protein (<50%; 50-90%; >90%)	15/12/20 (2 m)	10/11/33 (4 m)	16/22/25 (4 m)	0.89 (0.71-1.10)	0.28

HR= hazard ratio; CI = confidence interval; m = missing data

# n = 141 patients in the multivariate analysis;

* *p* ≤ 0.05

#### Younger patients with *IDH*-wildtype glioblastomas (*n* = 80)

Representing the younger population of primary glioblastomas, we used a TMA constructed from patients with high-grade gliomas aged 18-60 years. Patients were enrolled in a treatment cohort with RT or RT plus TMZ in various combinations, and 82 *IDH*-wildtype glioblastomas were selected from this TMA. Two samples with combined losses of 1p and 19q (1p19q codeletion) were excluded, leaving a total number of 80 tumors. Twelve patients received RT only as primary treatment, 68 patients received RT with TMZ. Table 2b shows the clinical and molecular characteristics of these 80 patients younger than 60 years with *IDH*-wildtype glioblastomas. Mean age was 51.4 years (range 24-60), mean survival was 26.5 months (range 0.5-129 months). At the time of last follow-up, 75 patients had died and 5 patients were alive. Multivariate analysis by Cox regression identified methylated *MGMT* promoter (*p* = 0.0326) and no steroids at baseline (*p* = 0.0070) as statistically significant favorable prognostic factors, while age (< 50 *versus* ≥ 50-60 years), gender and treatment (RT *versus* RT with TMZ) did not affect survival (Table 2b). WHO performance status (WHO 0-1, *n* = 73 *versus* 2, *n* = 7) and surgery (resection, *n* = 77 *versus* biopsy, *n* = 3) were not included in the model due to the uneven distribution of these parameters.

**Table 2b:** Multivariate Cox regression in primary glioblastomas for patients younger than 60 years (*n* =80)

Variables	*n*	HR# (95% CI)	*p*-value
Gender (female *vs* male)	32/48	0.98 (0.60-1.61)	0.94
Age (<50 *vs* 50-60)	23/57	0.99 (0.55-1.76)	0.96
Taking steroids at baseline (no *vs* yes)	56/24	0.48(0.29-0.82)	0.007*
MGMT status (methyl vs unmethyl)	39/38 (3 m)	0.58 (0.35-0.96)	0.033*
Treatment (RT *vs* RT+TMZ)	12/68	1.44 (0.70-2.98)	0.32
PROX1 protein (<50%; 50-90%; >90%)	23/42/15	1.13 (0.76-1.68)	0.53

HR= Hazard ratio; CI = confidence interval; m = missing data

# n = 77 patients in the multivariate analysis;

* *p* ≤ 0.05

#### Survival by PROX1 in younger patients with -wildtype glioblastomas

The distribution of PROX1 protein in the 80 *IDH*-wildtype glioblastomas from patients aged 18-60 years is shown in Table 2b. There was no statistically significant correlation between PROX1 expression and survival in the Cox regression model (*p* = 0.54) (Table 2b). There were no pairwise interactions between PROX1 and the other variables in the model.

### Selection of *IDH*-mutant astrocytomas grade III-IV (Table 3)

#### -mutant anaplastic astrocytomas and glioblastomas (*n* = 34)

**Figure 3:**
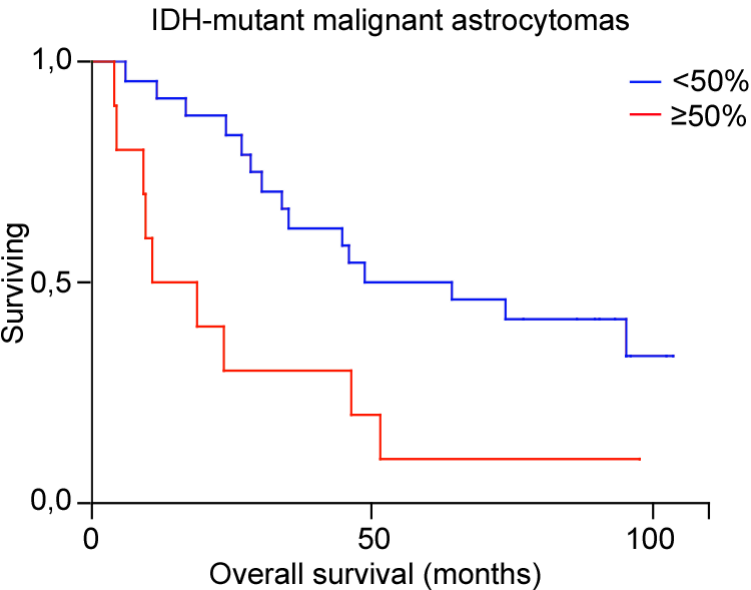
Kaplan Meier estimates of survival for 34 patients with *IDH*-mutant astocytomas grade III and IV included in the TMAs, with low (< 50%) *versus* high level (≥ 50%) of PROX1 protein

We then focused on the population of *IDH*-mutant anaplastic astrocytomas and glioblastomas. The TMA from patients aged 18-60 years comprised 19 *IDH*-mutant 1p19q non-codeleted high-grade astrocytomas, while the screening cohort comprised 15 high-grade astrocytomas that were immunopositive for mIDH1R132. The clinical characteristics of these 34 patients (20 *IDH*-mutant astrocytomas grade III, 14 *IDH*-mutant glioblastomas) are shown in Table 3. Mean age was 44.5 years (range 23-77), mean survival was 49.8 months (range 4.0-103.4). Because of the small sample size we used 50% as cut off value for PROX protein expression (instead of the 3-grading scale) in the statistical model. Univariate Kaplan-Meier model showed a statistically significant shorter survival for patients with tumors expressing high PROX1 protein (≥ 50% immunopositive tumor cells) (*p* = 0.009, Log-rank test) (Figure 3). The impact of PROX1 together with age and malignancy grade (WHO grade III *versus* IV) was analyzed by multivariate Cox regression (Table 3). Low PROX1 protein and younger age were associated with statistically significant longer survival. *MGMT* promoter methylation status was available for only 19/34 tumors and was not used in the model.

**Table 3:** Multivariate Cox regression in IDH-mutant anaplastic astrocytomas and glioblastomas (*n* = 34)

Variables	*n*	HR (95% CI)	*p*-value
Age		1.03 (1.00-1.07)	0.047*
Malignancy grade (WHO III *vs* IV)	20/14	0.81 (0.53-1.25)	0.34
PROX1 protein (< 50% *vs* ≥ 50%)	24/10	0.37 (0.16-0-94)	0.037*

### Validation by TCGA (Table 4)

#### -wildtype glioblastomas (*n* = 103)

To validate the findings for PROX1 in high-grade astrocytomas we used the Cancer Genome Atlas (TCGA) database (http://gliovis.bioinfo.cnio.es/). We selected *IDH*-wildtype glioblastomas and excluded tumors with 1p19q codeletion. Quantitative data for *PROX1* expression and survival data were collected for a total number of 103 patients (mean age 62.9 years, range 24-89 years; mean survival 11.4 months, range 0.2-47.6 months; dead/alive 77/26) (Supplementary Data, Table 1). Of these, 42 were *MGMT* promoter methylated, 61 had unmethylated *MGMT* promoter. Cases for which *MGMT* promoter methylation status was unknown were not included. Cox regression, using age and *PROX1* gene expression as continuous variables and *MGMT* promoter methylation status as dichotomized variable, identified age as an independent prognostic factor (*p* = 0.02) (Table 4a). No statistically significant pairwise interactions were found between *PROX1* and *MGMT* methylation status in the model.

**Table 4a:** Multivariate Cox regression in IDH-wildtype glioblastomas extracted from the Cancer Genome Atlas (*n*=103)

Variables	HR (95% CI)		*p*-value
Age	103	1.03 (1.00-1.05)	0.020*
MGMT status (methyl *vs* unmethyl)	42/61	0.67 (0.41-1.10)	0.11
PROX1 mRNA	103	0.90 (0.74-1.08)	0.25

HR= hazard ratio; CI = confidence interval;

* *p* ≤ 0.05

#### -mutant, 1p19q non-codeleted, astrocytomas grade III-IV (*n* = 110)

In the final step, we selected *IDH*-mutant astrocytomas grade III and *IDH*-mutant glioblastomas, excluding tumors with 1p19q codeletion. Thus, a total number of 110 *IDH*-mutant tumors (103 astrocytomas grade III and 7 glioblastomas) were included (mean age 38.1 years, range 21-76; mean survival 32.1, range 0-139 months; dead/alive 24/86) (Supplementary Data, Table 2). Of these cases, 99 had *MGMT* promoter methylation, 11 had unmethylated *MGMT* promoter. Cases with undetermined *MGMT* promoter methylation status were not included. Multivariate survival analysis by Cox regression, with age and *PROX1* entered as continuous variables and *MGMT* promoter methylation as dichotomized variable, identified *PROX1* gene expression as an independent prognostic factor (*p* = 0.0001), together with *MGMT* promoter methylation (*p* = 0.04) and WHO grade (*p* = 0.02), while age did not reach significance (*p* = 0.08) (Table 4b).

**Table 4b:** Multivariate Cox regression in IDH-mutant anaplastic astrocytomas and glioblastomas extracted from the Cancer Genome Atlas (*n*=110)

Variables		HR (95% CI)	*p*-value
Age	110	1.04 (1.00-1.07)	0.082
Grade (III *vs*. IV)	103/7	0.14 (0.04-0.68)	0.018*
MGMT status(methyl *vs* unmethyl)	99/11	0.19 (0.05-0.78)	0.040*
PROX1 mRNA	110	5.07 (1.07-13.06)	0.0001*

## DISCUSSION

In the present study we have unraveled the role of PROX1 as a prognostic factor for patients with high-grade astrocytomas. In three consecutive steps, we showed that PROX1 protein levels correlated with survival for patients with *IDH*-mutant high-grade astrocytomas but not for patients with primary glioblastomas. These findings were confirmed by *PROX1* gene expression analysis in tumors extracted from TCGA that were 1p19q non-codeleted and stratified by *IDH* mutation status. Together with our previous studies in low-grade gliomas, we conclude that PROX1 is a pathway-specific biomarker for *IDH*-mutant, 1p19q non-codeleted astrocytomas that may be used to identify high-risk patients. So far, there are few clinically useful biomarkers to predict individual outcome and guide clinical management for these patients [17]. Our results are of clinical importance and warrant the implementation of PROX1 in future trials of adults with *IDH*-mutant astrocytomas to further establish its role as a prognostic biomarker and potential treatment target.

The different cohorts that were used in this study are a strength but also a limitation of the present study. Our screening set consisted of a single-institution cohort of high-grade astrocytomas irrespective of *IDH* mutation status for which clinical data collection was performed post-hoc. The age-specific cohorts, on the other hand, were treatment cohorts of patients with glioblastomas enrolled in prospective clinical trials. DNA sequencing to verify that these were *IDH*-wildtype glioblastomas was performed for patients aged younger than 60 years, but not for patients aged 60 years and older enrolled in these trials. It is most likely that this cohort of elderly patients with newly diagnosed glioblastomas that lacked immunoreactivity for mIDH1R132 consisted of primary glioblastomas. However, the most recent WHO classification of brain tumors has reserved the diagnosis “glioblastomas NOS” for tumors for which full *IDH1/2* evaluation cannot be performed [5].

Due to the low number of *IDH*-mutant glioblastomas in our TMAs and in TCGA, we were not able to compare survival by PROX1 expression for patients with secondary glioblastomas separately. To obtain a representative group of *IDH*-mutant high-grade astrocytomas for PROX1 protein analysis, we collected the IDH1 immunopositive (mIDH1R132) and *IDH*-mutated 1p19q non-codeleted tumors in our TMAs. For studying *PROX1* gene expression in correlation to survival, we extracted *IDH*-mutant 1p19q non-codeleted anaplastic astrocytomas and glioblastomas from TCGA. The low number of *IDH*-mutant glioblastomas is consistent with the low incidence of secondary glioblastomas, comprising only around 10% of all glioblastoma cases [18]. In this context, it should be noted that the mean survival in the group of patients with *IDH*-mutant high-grade astrocytomas was significantly longer than in primary glioblastomas (32.1 months *versus* 11.4 months, tumors derived from TCGA). The negative results for PROX1 in the group of primary glioblastomas may therefore also exemplify the general difficulties to identify new biomarkers for patients with such a short survival time [19, 20].

Like other homeodomain containing proteins, PROX1 is able to both activate and repress transcription of genes in a context-dependent manner. The relationship between PROX1 and cancer is complex, and PROX1 can either exhibit tumor suppressing or oncogenic properties, depending on cancer type [7]. Interestingly, data extracted from TCGA displayed a statistically significant correlation between *PROX1* gene expression and survival for patients with *IDH*-mutant, 1p19q non-codeleted low-grade astrocytomas, but not with *IDH*-wildtype or 1p19q codeleted low-grade gliomas (Supplementary Figure 1). Thus, it seems that the prognostic impact of *PROX1* in low-grade gliomas is also pathway-dependent, consistent with our findings in high-grade astrocytomas. Whether PROX1 is a lineage-specific prognostic marker (i.e. for astrocytomas but not for oligodendrogliomas) is still unknown and needs to be studied further.

The diverting roles of PROX1 in *IDH*-mutant and *IDH*-wildtype astrocytomas may be related to different epigenetic regulatory mechanisms between these tumor types. *IDH*-wildtype glioblastomas predominate in elderly patients who have a generally higher degree of genomic methylation [21]. Promoter hypermethylation may be a regulatory mechanism for *PROX1* that is tumor type-specific, depending on cellular context and specific signaling pathways [22]. Indeed, *PROX1* was one of the identified target genes that were hypermethylated in a DNA screening of urine from patients with bladder cancer [23]. Aberrant DNA methylation of *PROX1* causing gene silencing has also been reported in hematological cancer [22]. A recent multi-platform genomic analysis of gliomas identified a subtype of *IDH*-mutant low-grade glioma that was associated with DNA demethylation and poor outcome [24]. It was also shown that tumors with high extent of genome-wide methylation could emerge as low methylated at recurrence, suggesting that DNA methylation is one of the mechanisms driving glioma progression [24].

High age is strongly associated with glioma incidence [25]. Age at disease onset is also one of the strongest single predictors for survival in all glioma types [13, 26]. It is largely unknown how aging affects glioma malignancy, but there is increasing evidence for fundamental molecular differences between high-grade gliomas of different age groups. Tumors of elderly patients with glioblastomas usually lack markers associated with favorable prognosis in the younger population [27]. It has been suggested that normal aging predisposes neural progenitor cells (NPCs) to increased malignant potential [28]. NPCs are tissue-specific progenitor cells that are the presumed cells of origin for gliomas [29,30], although differentiated cells such as astrocytes may also give rise to gliomas by dedifferentiation [31]. Age-related changes in the microenvironment of NPCs contribute to the increased cancer risk in the older brain [28]. The present study does not provide evidence for PROX1 as an age-dependent prognostic marker in primary glioblastomas. Instead, we found that PROX1 is a predictor for survival in the group of high-grade 1p19q non-codeleted astrocytomas that are *IDH*-mutant and predominate in younger patients with a prior history of low-grade gliomas.

In conclusion, the transcription factor PROX1, with a widespread role in CNS development, is a prognostic marker for *IDH*-mutant high-grade astrocytomas that evolve through multi-step genetic alterations over time. Together with our previous study in low-grade gliomas [12], we propose that PROX1 is a clinically valuable biomarker to predict the course of disease for these patients. Future studies are needed to clarify the mechanisms by which PROX1 leads to tumor progression and affects clinical outcome in this patient group.

## MATERIALS AND METHODS

### Patient cohorts

#### Screening cohort of unselected high-grade astrocytomas

For PROX1 protein screening we selected 86 astrocytomas WHO grade III and IV from a TMA of a previously described retrospective cohort of 96 adult patients with high-grade gliomas [15]. Oligodendroglial tumors were excluded based on histological tumor diagnosis. Mutated IDH1 (R132H) protein was detected by immunohistochemistry. Molecular characterization for detection of *IDH1/2* mutations, 1p19q codeletions and *MGMT* promoter methylation status was not performed. All patients received RT, concomitant and adjuvant TMZ was not used consistent with standard protocols at that time. Thirty-one patients received chemotherapy and 19 patients had proton radiation as second-line treatment [15]. Survival was defined as the time point between surgery and date of death. All patients had died at the end of the study. Data concerning time of death and the cause of death were collected from central health authorities. Tissues were obtained and used in a manner compliant with the Declaration of Helsinki. The local ethical committee approved the study protocol (Application Dnr Ups 02-330).

#### Cohort of elderly patients with newly diagnosed glioblastoma

In the context of the Nordic trial for patients aged 60 years or older with glioblastoma, a TMA was constructed as a powerful tool to screen candidate markers for response to therapy or outcome in this trial [14]. The TMA comprised 175 paraffin embedded glioblastoma tissues from patients enrolled in the trial divided over three treatment-arms; standard RT 60 Gy (*n* = 49), hypofractionated RT 34 Gy (*n* = 58) and temozolomide (*n* = 67). Study treatment was started in 65 of the 67 patients in the temozolomide group, 46/49 patients in the standard RT group, and 57/58 patients in the hypofractionated RT group. Second-line treatment, where reported, in the temozolomide group was: 9 chemotherapy, 27 RT, 4 re-operation, 1 bevacizumab and 1 imatinib, in the hypofractionated RT: 20 chemotherapy, 1 re-radiation and 1 re-operation, and in the 60 Gy RT group: 18 chemotherapy, 1 re-radiation, 2 re-operation and 2 bevacisumab. The TMA was constructed with an agarose matrix at the Service of Neurosurgery, Lausanne University Hospital, Switzerland [31]. Mutated IDH1 (R132H) protein was detected by immunohistochemistry on TMA or whole tumor sections. Molecular characterization of *MGMT* promoter methylation status was performed, but not of *IDH1/2* mutations and 1p19q codeletions.

#### Cohort of younger patients with primary glioblastoma

To represent the younger population of glioblastomas, we used a TMA from patients aged 18-60 years with high-grade gliomas enrolled in a treatment cohort with RT or RT and TMZ in various combinations. Inclusion criteria were histologically proven astrocytoma grade III or IV and age 18-60 years. A total of 145 patients were included between 2003-2008 and 110 tumor samples were available for construction of TMA and DNA extraction. Molecular characterization included analysis of 1p19q codeletion, *MGMT* promoter methylation status and *IDH1/2* mutations. The present study describes the results for 80 patients with *IDH*-wildtype glioblastomas, and for 19 additional patients with *IDH*-mutant astrocytoma grade III or IV that were included in this TMA. Data collection included molecular tumor markers, histological diagnosis, age, treatment arm (RT *versus* RT plus TMZ), type of surgery, WHO performance status, steroids at baseline, and overall survival.

### Immunohistochemistry

Immunohistochemical staining and slide scanning was performed in accordance with strategies used in The Human Protein Atlas project (www.proteinatlas.org) [33]. Immunostainings were performed at the SciLifeLab Tissue Profiling Facility, Uppsala University, Sweden [34]. Automated immunohistochemistry was performed as previously described using a LabVision Autostainer 480S (Thermo Fisher Scientific, Runcorn, UK) [35].

#### Antibodies

Immunostaining for supportive verification of the histopathological diagnosis included diagnostic antibodies directed to glial fibrillary acidic protein (GFAP) (1:500; polyclonal rabbit, DAKO, Denmark), microtubule-associated protein 2 (MAP2) (1:100; mouse clone HM2, Sigma), Ki67 (1:500; rabbit polyclonal, DAKO), and mutated isocitrate dehydrogenase 1 (IDH1) R132H protein (1:100; monoclonal mouse antibody, mIDH1R132) [16]. For detection of PROX1 we used rabbit anti-PROX1 (HPA000385; 1:100 dilution) [12].

#### Evaluation of immunohistochemistry

At least two observers (TE, JC, KRR, AS) independently evaluated the immunostainings and assessed the proportion of labeled tumor cells in each sample. The entire section of microtissue was examined. In case of diversities (approximately 15% of all evaluated samples in this study), the scoring results were re-evaluated by the two observers to reach consensus. The fraction of labeled tumor cells was graded manually into a 3-grade scale: 1 (+) = < 50% strong positive tumor cells; 2 (++) = ≥ 50% and < 90% strong positive tumor cells; 3 (+++) = ≥ 90% strong positive tumor cells. Clear and distinct immunoreaction was considered as positive staining, while faint and less distinct immunoreaction was considered negative.

### Molecular analyses

#### MGMT promoter methylation

A modified pyrosequencing method published by Collins et al. was used to assess MGMT methylation status [36]. After bisulfite modification of 50-200 ng of DNA using EZ DNA Methylation Kit (Zymo Research), nested PCR was carried out with HotStarTaq Master Mix (Qiagen), annealing temperature of 51°C and primers: forward primer, primary PCR TTTAYGTYGTTATTTTTGTGTTTTT, forward primer, secondary PCR GTTTYGGATATGTTGGGATAG, reverse primer used in both reactions biotin-AAAACCACTCRAAACTACCAC. Obtained PCR product was used as a template in 4 pyrosequencing assays. In brief, per one assay 15 μl of the PCR product was mixed with 40 μl of binding buffer, 23 μl of water and 2 μl of sepharose beads (GE Healthcare). Biotinylated DNA strand bound to the beads was isolated with a Vacuum Prep Workstation (Qiagen) and moved to the 15 μl of mixture containing 0.3 μM sequencing primer in the annealing buffer. Sequencing primer was annealed to the template at 80°C for 2 min. Pyrosequencing was performed on a PyroMark Q96 MD instrument (Qiagen) using PyroMark Gold Q96 CDT Reagents (Qiagen). Sequencing primers, sequences to analyze and dispensation orders for each pyrosequencing assay were as follows: amplicon1- GATAGTTYGYGTTTTTAGAA, YGTTTTGYGTTTYGAYGTTYGTAGGTTTT, ATCGTTCAGTCTGTTCGTATCAGTCGTCA; amplicon2-TTTYGAYGTTYGTAGGTTT, YGYGGTGYGTATYGTTTGYGA, GTCTGTCGTAGTCGTGATCGTAGTCGA; amplicon3-GYGATTTGGTGAGTGTTTG, GGTYGTTTYGTTTTYGGAAGAGTGYGG, AGTCTGTTCAGTTCGAGAGTAGTCG; amplicon4- GAAGAGTGYGGAGTTTTTTTT, YGGGAYGG, ATCGTATCG. Samples were run in duplicates and triplicates. Pyrosequencing data were analyzed using Pyro Q-CpG software version 1.0.9, giving % methylation for each CpG site. Mean values for each site from different runs were used to calculate the mean methylation value for the entire region. Samples with a mean value of methylation from all 16 CpGs < 9% were considered unmethylated and ≥ 9% as methylated, this cut-off providing the most significant survival difference among patients with *IDH*-wildtype tumor.

#### Mutation analysis of IDH

IDH mutation status was confirmed by pyrosequencing. In the pyrosequencing assay a 108bp long PCR product was amplified using following primers: biotin-CAAAAATATCCCCCGGCTTG and ACATGCAAAATCACATTATTGCC in the concentration of 1 μM each mixed with 1 U MyTaq DNA polymerase (Bioline), 5 mM MyTaq Buffer and 20 ng of template DNA in a final volume of 20 μl. The annealing temperature was 57°C. The confirmation of successful amplification was done by 1,5% agarose gel electrophoresis. Single-stranded template was prepared as described above and annealed to the sequencing primer ACTTACTTGATCCCCATAAGCAT. For the following sequence to analyze GA[T/C]GACCTATGATAGGTTTTACCCA, dispensation order of CGATCTCGAC was used. Reagents and instrument were used as previously mentioned. Data were analyzed using PyroMark MD 1.0 software.

#### Evaluation of 1p19q codeletion

Codeletion on chromosome 1p19q was investigated using droplet digital PCR. The method was based on the detection of changes in the copy numbers of genes located on arms 1p and 19q (1p13.2 LRIG2, 1p31.1 FUBP1, 1p32.3 CDKN2C and 19q12 CCNE1, 19q13.11 CEBPA, 19q13.32 ERCC1) using the QX100 Droplet Digital PCR system (BioRad). In case of loss of one copy in all of the genes, 1p19q codeletion was stated. Samples were subjected to six reactions, each dedicated to a specific gene. Ten ng of the DNA isolated from paraffin sections was mixed with 11 μl of ddPCR Supermix for Probes (BioRad), 1.1 μl of the probe for the gene of interest and 1.1 μl of the probe for the reference gene in the final volume of 22 μl. Manufacturer's recommendations for the preparation of droplets and the PCR were applied on all of the genes except FUBP1, where the annealing temperature was raised to 61°C. The following probes have been used: CDKN2C (dHsaCP1000589, BioRad), CEBPA (dHsaCP1000494, BioRad) with reference AP3B1 (dHsaCP2500348) and FUBP1 (Hs05722030, Applied Biosystems), LRIG2 (Hs06588310, Applied Biosystems), CCNE1 (Hs01813172, Applied Biosystems) and ERCC1 (Hs01171620, Applied Biosystems) with reference AP3B1 (dHsaCP1000001, BioRad), what was dictated by the differences in the length of the probes. Probes for the genes were labelled with FAM dye and for the reference gene with HEX dye. Data was analyzed using the QuantaSoft software v. 1.2.10 (BioRad) and automated clustering analysis [37].

### The Cancer Genome Atlas

To confirm the results obtained by the TMAs on the correlation between PROX1 expression and patient survival we extracted data from TCGA (http://gliovis.bioinfo.cnio.es/) (last update 8^th^ May 2016). We included all astrocytomas WHO grade III and glioblastomas for which comprehensive data on *PROX1* transcriptome, *IDH* mutation status, *MGMT* promoter methylation status, survival time and patient age were available. Tumors harboring codeletions on chromosomes 1p and 19q were excluded. Two separate datasets, one comprising 110 *IDH*-wildtype glioblastomas (TCGA Dataset IDH-wildtype, Supplementary Table 1) and one comprising 103 *IDH*-mutant astrocytomas grade III/IV (TCGA Dataset IDH-mutant, Supplementary Table 2) were analyzed. For survival analysis PROX1mRNA and patient age were used as continuous variables, and *MGMT* promoter methylation status as dichotomized variable.

### Statistical analysis

Survival curves were plotted according to the Kaplan-Meier method (product-limit method) and the log-rank probability test estimated the prognostic value of PROX1 and other variables by univariate analysis. Multivariate Cox regression was used to calculate hazards ratios (HR) for relative risk of death. We tested for pairwise interactions between PROX1 and the variables *MGMT* promoter methylation, *IDH* mutation and patient age. Statistical analysis was performed in JMP, version 10.0 (SAS Institute Inc., Cary, North Carolina, USA) and in SPSS, version 22.

## SUPPLEMENTARY MATERIALS TABLES FIGURE






