# Supplementary material for: PROX1 is a novel pathway-specific prognostic biomarker for high-grade astrocytomas; results from independent glioblastoma cohorts stratified by age and IDH mutation status
Source: Oncotarget. 2016 Sep 10;7(45):72431–42. doi: 10.18632/oncotarget.11957 (PMC5341919; doi:10.18632/oncotarget.11957)
Supplement: Supplementary file 1 [file oncotarget-07-72431-s001.pdf]

PROX1 is a novel pathway-specific biomarker for high-grade astrocytomas; results from independent glioblastoma cohorts stratified by age and *IDH* mutation status

Supplementary Material

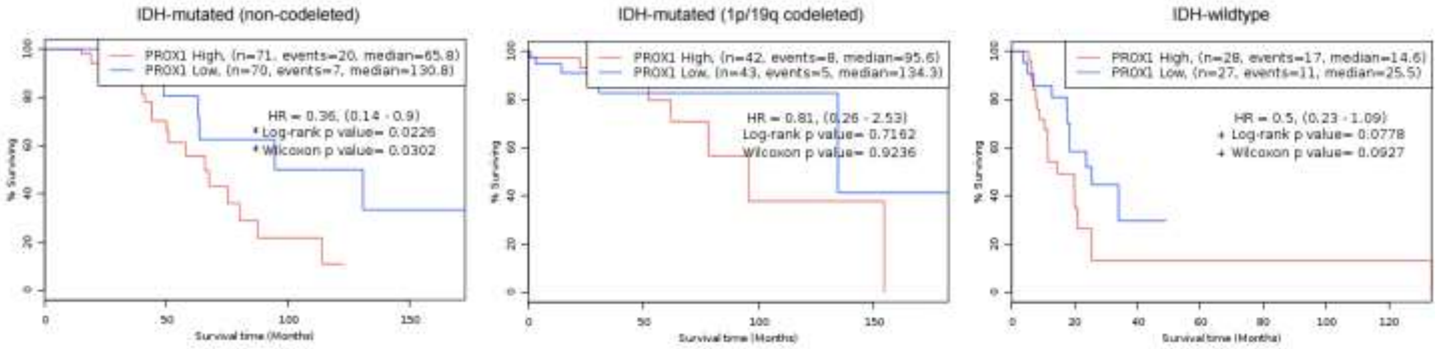

Kaplan-Meier estimates for survival by PROX1 gene expression in low-grade gliomas separated by subtype. Plots are reproduced from http://gliovis.bioinfo.cnio.es/ and represent data extracted from the TCGA database.

For Data Table S1, please see the attached Excel file

For Data Table S2, please see the attached Excel file
